# A plea for personalization of the hemodynamic management of septic shock

**DOI:** 10.1186/s13054-022-04255-y

**Published:** 2022-12-01

**Authors:** Daniel De Backer, Maurizio Cecconi, Michelle S. Chew, Ludhmila Hajjar, Xavier Monnet, Gustavo A. Ospina-Tascón, Marlies Ostermann, Michael R. Pinsky, Jean-Louis Vincent

**Affiliations:** 1 Department of Intensive Care, CHIREC Hospitals, Université Libre de Bruxelles, Boulevard du Triomphe 201, 1160 Brussels, Belgium; 2 Humanitas Clinical and Research Center – IRCCS, Rozzano, MI Italy; 3 Department of Biomedical Sciences, Humanitas University, Pieve Emanuele, MI Italy; 4 Department of Anaesthesia and Intensive Care, Biomedical and Clinical Sciences, Linköping University, Linköping, Sweden; 5 Departamento de Cardiopneumologia, InCor, Faculdade de Medicina da Universidade de São Paulo, São Paulo, Brazil; 6 AP-HP, Service de Médecine Intensive-Réanimation, Hôpital de Bicêtre, DMU 4 CORREVE, Inserm UMR S_999, FHU SEPSIS, CARMAS, Université Paris-Saclay, 78 Rue du Général Leclerc, 94270 Le Kremlin-Bicêtre, France; 7 Department of Intensive Care, Fundación Valle del Lili, Cali, Colombia; 8 Translational Research Laboratory in Critical Care Medicine (TransLab-CCM), Universidad Icesi, Cali, Colombia; 9 Department of Intensive Care, King’s College London, Guy’s & St Thomas’ Hospital, London, UK; 10 Department of Critical Care Medicine, University of Pittsburgh, Pittsburgh, PA USA; 11 Dept of Intensive Care, Erasme Univ Hospital, Université Libre de Bruxelles, Brussels, Belgium

**Keywords:** Blood pressure, Cardiac output, Tissue perfusion, Fluids, Vasopressor agents, Inotropic agents

## Abstract

Although guidelines provide excellent expert guidance for managing patients with septic shock, they leave room for personalization according to patients’ condition. Hemodynamic monitoring depends on the evolution phase: salvage, optimization, stabilization, and de-escalation. Initially during the salvage phase, monitoring to identify shock etiology and severity should include arterial pressure and lactate measurements together with clinical examination, particularly skin mottling and capillary refill time. Low diastolic blood pressure may trigger vasopressor initiation. At this stage, echocardiography may be useful to identify significant cardiac dysfunction. During the optimization phase, echocardiographic monitoring should be pursued and completed by the assessment of tissue perfusion through central or mixed-venous oxygen saturation, lactate, and carbon dioxide veno-arterial gradient. Transpulmonary thermodilution and the pulmonary artery catheter should be considered in the most severe patients. Fluid therapy also depends on shock phases. While administered liberally during the resuscitation phase, fluid responsiveness should be assessed during the optimization phase. During stabilization, fluid infusion should be minimized. In the de-escalation phase, safe fluid withdrawal could be achieved by ensuring tissue perfusion is preserved. Norepinephrine is recommended as first-line vasopressor therapy, while vasopressin may be preferred in some patients. Essential questions remain regarding optimal vasopressor selection, combination therapy, and the most effective and safest escalation. Serum renin and the angiotensin I/II ratio may identify patients who benefit most from angiotensin II. The optimal therapeutic strategy for shock requiring high-dose vasopressors is scant. In all cases, vasopressor therapy should be individualized, based on clinical evaluation and blood flow measurements to avoid excessive vasoconstriction. Inotropes should be considered in patients with decreased cardiac contractility associated with impaired tissue perfusion. Based on pharmacologic properties, we suggest as the first test a limited dose of dobutamine, to add enoximone or milrinone in the second line and substitute or add levosimendan if inefficient. Regarding adjunctive therapies, while hydrocortisone is nowadays advised in patients receiving high doses of vasopressors, patients responding to corticosteroids may be identified in the future by the analysis of selected cytokines or specific transcriptomic endotypes. To conclude, although some general rules apply for shock management, a personalized approach should be considered for hemodynamic monitoring and support.

## Introduction

Shock is a life-threatening condition characterized by inadequate delivery of oxygen to tissues [1]. Septic shock is one of the most common causes of shock in the ICU [2]. The Surviving Sepsis Campaign (SSC) Guidelines provide excellent guidance for the management of septic patients [3], but for several reasons there is room for personalization [4]. First, although these guidelines are supported by evidence, they are based primarily on randomized controlled trials (RCTs) investigating the response of large groups of patients to an intervention. These trials are generally negative, i.e., do not reveal differences in mortality. It is important to recognize that individual patient specificities may affect the response or tolerance to a given intervention. Second, many areas of resuscitation are still a matter of debate and research gaps remain [5]. Hence, guidelines often fail to offer strong and precise recommendations in specific areas. Third, there are different phases in the management of shock [2], each requiring a different approach. Thus, it may be justified to individualize the therapeutic options according to the patient’s condition. In this expert opinion paper, we discuss the different options regarding the personalization of hemodynamic monitoring and management of septic shock patients, at the various stages of shock. The general principle of personalized shock management is to measure, interpret, apply therapy, evaluate its effects, and react, in contrast to applying standard measures.

## Personalization of monitoring

**Fig. 1:**
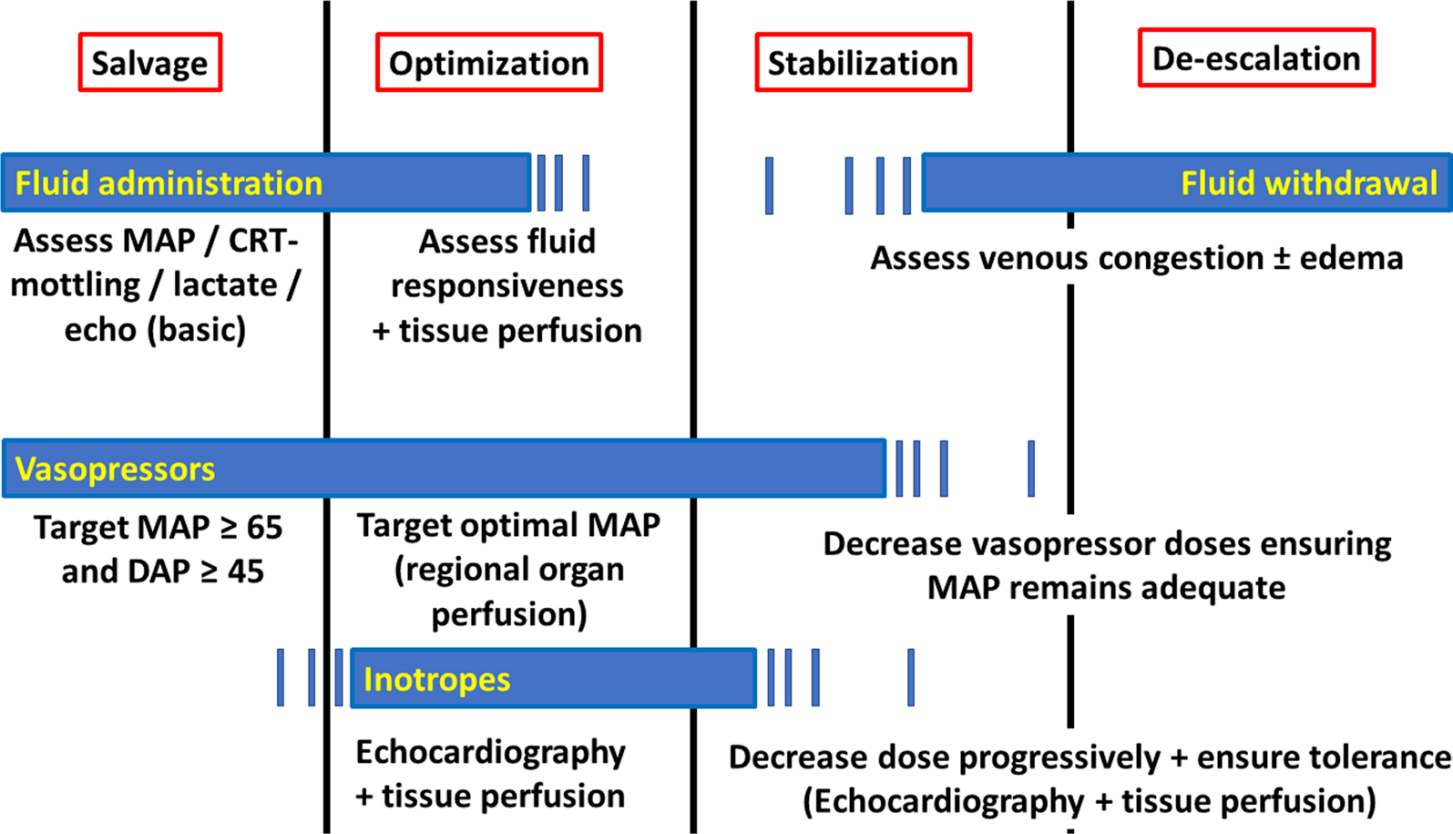
Suggested monitoring and interventions at the different stages of shock. Therapeutic options (yellow in blue rectangles) and monitoring techniques and goals at the different stages of septic shock. MAP mean arterial pressure, CRT capillary refill time, echo echocardiography, DAP diastolic blood pressure

Personalization of hemodynamic monitoring implies considering the different SOSD phases (salvage, optimization, stabilization, and de-escalation). At each stage, the available techniques and targets for resuscitation vary (Fig. 1 and Table 1).

**Table 1:** Targets and monitoring techniques at the different phases of shock

Phase of shock	Purpose	Targets	Interventions	Monitoring tools
Salvage	*Perform life-saving measures	*Maintain minimal MAP and CO	*Fluids *Vasopressors according to MAP and DAP	*Arterial pressure (often noninvasive, turn to invasive if not responding) *CRT *Lactate
	*Identify shock			*Clinical examination *Arterial pressure *Lactate
	*Identify severe cardiac dysfunction			*Echocardiography *CRT *Jugular vein distension
Optimization				
*Initial*	*Optimize tissue perfusion	*Normalize indices of tissue perfusion *Optimize MAP *Optimize CO	*Fluids according to fluid responsiveness and tolerance *Vasopressors *Inotropes according to CO and echocardiography	*CRT *Lactate *CVP – ScvO2-PvaCO2 *Urine output *Minimally invasive CO *Echocardiography if not yet performed *Evaluation of fluid responsiveness
*Subsequent (if not responding to initial)*	*Optimize tissue perfusion	*Normalize indices of tissue perfusion *Optimize MAP *Optimize CO	*Fluids according to fluid responsiveness and tolerance *Vasopressors *Inotropes according to CO and echocardiography	*CRT *Lactate *CVP – ScvO2-PvaCO2 *Urine output *Evaluation of fluid responsiveness *Advanced hemodynamic monitoring (TPTD or PAC ± Echocardiography) *Venous ultrasounds
Stabilization	*Provide organ support *Minimize complications	*Preserve organ perfusion *Limit exposure to fluids, vasopressors and inotropes, if possible *Limit impact of accumulated fluids	*Avoid fluids unless absolutely needed, if possible consider fluid removal *Lowest dose of vasopressors to achieve target MAP *Lowest dose of inotropes to maintain target CO	*Maintain existing monitoring *EVLW (TPTD or lung ultrasound) *Venous stasis
De-escalation	*De-escalate engaged therapies while avoiding impairment in tissue perfusion	*Achieve negative fluid balance *Wean vasopressors *Wean inotropes *Preserve tissue perfusion	*Fluid removal by diuretics and/or mechanical *Wean vasopressors if MAP preserved *Wean inotropes if acceptable CO preserved	* Arterial pressure (often noninvasive) *Minimal or no CO-monitoring at this stage *Evaluate fluid responsiveness prior to fluid removal *Evaluate tissue perfusion *Lactate

*MAP* mean arterial pressure, *DAP* diastolic arterial pressure, *CO* cardiac output, *CRT* capillary refill time, *CVP* central venous pressure, *ScvO2* central venous pressure, *PvaCO2* veno-arterial difference in *PCO2*, EVLW extravascular lung water, *TPTD* transpulmonary thermodilution, *PAC* pulmonary artery catheter

### Time of shock recognition: salvage phase

At the time of shock recognition and identification, the initial treatment aims to provide tissue perfusion levels compatible with life. In this phase, hemodynamic monitoring is often very basic and therapeutic options limited to fluids and vasopressors.

#### What information may be derived from basic hemodynamic monitoring in guiding resuscitation?

Clinical evaluation is crucial to identify tissue hypoperfusion. Blood pressure measurement, skin mottling, and capillary refill time (CRT) inform on the progress of resuscitation. Alterations in CRT and mottling scores can be used for basic monitoring as they correlate with outcome [6, 7] and rapidly respond to therapeutic interventions. However, they are poorly correlated with cardiac output (CO), do not identify the source of tissue hypoperfusion, and cannot direct the necessary interventions [8].

If medical history suggests that cardiac function may be impaired or if the patient fails to respond to fluids, rapid echocardiographic evaluation is useful to refine the hemodynamic evaluation [1]. Bedside echocardiography is the sole technique that allows a rapid estimation of CO along with the identification of the cause of low CO. In a recent RCT, echocardiography use by the rapid response team was associated with improved survival [9].

During salvage, inotropes may be indicated when a relevant cardiogenic component (related to septic cardiomyopathy or patient comorbidities) contributes to the shock process [1, 10]. This can only be identified by echocardiography, and inotropes should be considered when CO is low due to severely impaired cardiac contractility. In severe cardiogenic shock component, echocardiography can also rapidly identify patients not responding to initial therapy and for whom mechanical cardiac support may be considered [11].

Basic clinical monitoring has a role in identifying patients who may respond to fluids and assessing their response [12]. In particular, CRT can be used to evaluate the tissue perfusion response and to guide the need on requirements for further fluids administration [13]. Dynamic tests such as the response in pulse pressure or CRT to a passive leg raising can be used to predict fluid responsiveness [14, 15]. However, these tests are difficult to perform during the salvage phase when many interventions are applied simultaneously. Prediction of fluid responsiveness and assessment of the effects of fluids should be undertaken as soon as technically feasible, especially in patients with poor cardiac function.

Measuring blood lactate levels is useful to identify impairment in tissue perfusion. In addition, when a central venous catheter is in place, central venous O_2_ saturation (ScvO_2_) and the gradient of carbon dioxide partial pressure (pCO_2_) between central venous and arterial blood (Pv-aCO_2_) may be useful to guide resuscitation.

#### How to identify patients who benefit from immediate vasopressor therapy without waiting for the effects of fluid resuscitation?

While some patients may respond to fluid therapy alone, others need vasopressor support. The SSC guidelines recommend maintaining mean arterial pressure (MAP) ≥ 65 mmHg but do not indicate timing or provide guidance on prioritization of fluids versus vasopressors. Some patients with severe hypotension may require starting vasopressors early, i.e., without waiting for the fluid effects, in order to accelerate the restoration of arterial pressure. Indeed, delaying the correction of hypotension is associated with poor outcome [16]. Moreover, norepinephrine may contribute to increasing cardiac preload by recruiting unstressed blood volume [17], which may reduce fluid requirements. In an observational study using propensity matching, early start of norepinephrine was associated with a less positive fluid balance and lower 28-day mortality [18].

The decision to start vasopressors early, along with fluid therapy, seems logical in cases of profound hypotension, although no precise cutoff of MAP can be given. Diastolic pressure (DAP) may also guide the decision. DAP is determined by vascular tone and the decay time of aortic blood volume. In sepsis, a low DAP often reflects severe vasodilation and is associated with increased mortality [19]. Then, it seems logical to initiate vasopressors when DAP is very low, e.g., < 45 mmHg. In tachycardic patients, high values of diastolic shock index (DSI), the ratio between DAP and HR, are associated with a higher risk of death in patients with septic shock [19]. Whether DSI > 2 should be used to trigger vasopressors remains unknown.

### Optimization phase: tools and targets

At this stage, the goal of resuscitation is to optimize tissue perfusion through optimization of perfusion pressure and CO. Ideally, this should also include optimization of regional blood flow distribution and microcirculatory perfusion. While alterations in the distribution of regional blood flow [20] and microvascular perfusion [21] are frequent in shock and may be dissociated from the systemic circulation, they cannot be monitored easily in clinical practice.

The most frequent indices of tissue hypoperfusion used at the bedside include arterial pressure, urine output, skin perfusion, CRT, ScvO_2_, Pv-aCO_2_, and lactate concentrations. Importantly, even if some correlation between the different indices exists at baseline, some variables normalize more rapidly than others [22]. ScvO2 seems to normalize most rapidly followed by CRT and Pv-aCO2 which usually normalize within 6–8 h, while lactate and sublingual microcirculation may take more than 24 h to normalize [13]**.** It seems logical to combine several variables and to stop resuscitation when most are normalized, without pursuing normalization of the variables like lactate concentrations that have a longer lag time [23]. When tissue hypoperfusion is detected, fluid responsiveness should be assessed and, in some cases, advanced hemodynamic techniques should be considered for the evaluation of cardiovascular function.

#### Capillary refill time

CRT is a marker of skin hypoperfusion that dynamically responds to vasoactive substances. The extent to which CRT reflects central tissue perfusion remains unknown. While one study concluded that CRT reflected central circulation [24], other trials suggested some dissociation [22, 25]. CRT measurements show significant interobserver variability [26], but this can be minimized with the standardization of the technique [13]. When compared to lactate-targeted resuscitation in early septic shock, CRT-guided resuscitation tends to be superior [13, 27]. Among the factors contributing to these differences are more frequent assessments of the circulation in the CRT group. Reaching CRT values < 3 s is a valuable guide to resuscitation, whereas the slow decrease in lactate levels alone may result in additional fluid administration even though tissue perfusion may have normalized at the time of assessment.

#### Mean arterial pressure

Determining the systemic blood pressure target necessary to achieve adequate tissue perfusion remains difficult. In principle, organ blood flow depends on perfusion pressure (i.e., difference between inflow and outflow pressure) and resistance. However, preservation of systemic arterial pressure is not sufficient to ensure the adequacy of microcirculatory flow. Importantly, perfusion pressures differ across vascular beds (Table 2), and those levels may also be affected in sepsis.

**Table 2:** Organ-specific perfusion pressures and predominant vasoconstrictor receptors

Organ	Perfusion pressure	Predominant vasoconstrictor receptors
Brain	MAP–(CVP or ICP)*	α1, α2, angiotensin, vasopressin, endothelin, dopamine, neuropeptide Y, endothelial purinergic, 5-HT1B, endothelial purinergic
Heart	DAP–(CVP or ITP)*	α1, α2, angiotensin, vasopressin, endothelin, thromboxane, dopamine
Kidney	MAP–(CVP or IAP or IRP)*	α1, α2, angiotensin, vasopressin, endothelin, sphingosine-specific G protein-coupled
Gut	MAP–(CVP or IAP)*	α1, α2, angiotensin, vasopressin, endothelin, neuropeptide Y, constrictor prostaglandins
Liver	Hepatic artery: MAP–(CVP or IAP)* Portal vein: portal vein pressure—(CVP or IAP)*	α1, α2, angiotensin, vasopressin, endothelin, COX1-derived prostanoids, thromboxane

DAP = diastolic arterial pressure; CVP = central venous pressure; HT1B = hydroxytryptamine receptor 1B;

IAP = intra-abdominal pressure; ITP = intrathoracic pressure, IRP = intrarenal pressure (the kidney is surrounded by a non-distensible membrane and sensitive to interstitial edema)

*Whatever pressure is higher

Most organs have the endogenous ability to preserve the microcirculation within a certain range of perfusion pressures. For instance, heart and brain can maintain a constant blood flow despite large changes in perfusion pressure. In case the pressure falls below the organ-specific autoregulation zone, organ blood flow becomes dependent on inflow pressure, hence the focus on reversing hypotension during the salvage state.

The heterogeneity of patients, distinct organ-specific microcirculatory regulation, variable receptor density, and the impact of pharmacological interactions make a uniform approach to septic shock challenging [28]. The SSC Guidelines recommend an initial MAP target of 65 mmHg [3] but include no recommendations for later stages. Observational data suggest that organ dysfunction may sometimes already begin when MAP falls below 75–80 mmHg [16], but reaching higher MAP targets often requires higher doses of vasopressors which may be associated with more adverse events. Trials randomizing septic patients to MAP targets ~ 65 mmHg or even less compared to ~ 75 and ~ 85 mmHg showed no difference in mortality [29, 30]. A beneficial impact of higher MAP on renal function in previously hypertensive patients can be observed [29] but is not systematic [30]. Differences in the effect of higher MAP targets on renal function may in part be explained by a high variability in the response in renal blood flow to an increase in MAP [31]. The failure of the “one size fits all” approach was also demonstrated in patients with hypotension. An analysis of 3542 critically ill patients with shock showed that patients with naturally low BP (systolic BP < 100 mmHg) were treated for longer and with higher doses of norepinephrine and had a longer ICU stay and higher mortality [32]. Accordingly, while an initial MAP target of 65 mmHg seems a reasonable approach for many patients, the ideal MAP should be individualized and ideally be based on a MAP challenge. The MAP challenge consists in the evaluation of changes in perfusion indices such as urine output, level of consciousness, and cutaneous perfusion during a transient increase in MAP. If beneficial effects are observed, this new MAP target can be used, if inefficient or not tolerated then the initial MAP value should be targeted.

#### Central venous pressure (CVP)

CVP is a complex variable, reflecting right ventricular preload and function when elevated, and affected by intrathoracic pressures [33]. Although its ability to precisely predict the response to fluids is challenged, it still provides important information on fluid status and right ventricular reserve and should be measured in shock [34]. CVP may be an important early indicator of the failing right ventricle. A sustained elevated CVP > 12 mmHg is associated with impaired renal and gut function even when organ perfusion pressure is held constant, suggesting that venous congestion plays a role in organ dysfunction. There should be no target value for CVP, as the ideal CVP is the lowest CVP associated with hemodynamic stability.

#### Cardiac output

CO is a key determinant of tissue perfusion. However, there is no fixed value of optimal CO in shock and CO should be optimized according to tissue perfusion indices listed below and organ function. Importantly, microvascular alterations may persist even when a low CO is corrected, impairing tissue perfusion [21, 35]. In hyperkinetic shock, the persistent perfusion abnormalities are attributable to alterations in regional perfusion and/or alterations in microvascular perfusion. These microvascular alterations may be insensitive to an increase in CO, and other strategies should be used to improve the microcirculation. Accordingly, CO should be considered as a means to improve tissue perfusion and not as a target.

#### Mixed-venous (SvO_2_) and central venous (ScvO_2_) oxygen saturation

Although ScvO_2_ or SvO_2_ is not even quoted in the new SSC guidelines [3], they are very important physiologic variables that need to be understood and measured. They reflect the balance between the actual oxygen consumption and tissue oxygen delivery. Accordingly, a low ScvO_2_ indicates impaired or inadequate O_2_ delivery, explained by an inadequate CO if hemoglobin and arterial O_2_ saturation are within normal ranges. In sepsis, SvO_2_ and ScvO_2_ are expected to be normal or elevated even when tissue perfusion is impaired [21]. While targeting specific SvO_2_ or ScvO_2_ values in all patients has been challenged [36], finding a low SvO_2_ or ScvO_2_ may identify patients who should benefit from further resuscitation efforts. This may consist in increasing CO with fluids or an inotropic agent, or sometimes Hb by transfusing blood in anemic patients. In patients with high ScvO2, other indices of impaired perfusion should be carefully checked.

#### Venous-to-arterial carbon dioxide difference (Pv-aCO_2_)

Pv-aCO_2_ depends on the total carbon dioxide (CO_2_) production, CO and microvascular perfusion, and the complex relationship between CO_2_ partial pressures and CO_2_ blood contents. According to the modified Fick equation, Pv-aCO_2_ is inversely related to CO (curvilinear relationship). Under stable conditions of both oxygen consumption (VO_2_) and CO_2_ production (VCO_2_), Pv-aCO_2_ progressively increases in response to reductions in CO due to the CO_2_-stagnation phenomenon in microvessels. Progressive increases in Pv-aCO_2_ reflect decreased microcirculatory perfusion in the early stages of septic shock [37]. Thus, a high Pv-aCO_2_ may identify septic patients who are inadequately resuscitated [38]. Admission of Pv-aCO_2_ values [39] and changes in Pv-aCO_2_ in response to therapy are both associated with outcome [40]. Measuring Pv-aCO_2_ may be particularly helpful in patients with normal values of lactate or ScvO_2_, identifying those at risk of poor outcome despite apparent adequate resuscitation [40–42]. It is nevertheless difficult to recommend a specific Pv-aCO_2_ target based on observational trials. The ratio of Pv-aCO_2_ over the arteriovenous difference in oxygen content is an estimate of the respiratory quotient and, as such, may be a direct indicator of anaerobic metabolism, with faster changes than lactate [42, 43].

#### Blood lactate concentrations

Lactate levels have a strong prognostic value and can thus be used for triage. The assessment of serial lactate levels is useful, as these usually decrease in patients who improve and often remain elevated or even increase when septic shock is poorly controlled. Resuscitation strategies targeting decreases in lactate level were associated with decreased hospital mortality [44]. Thus, it seems logical to guide resuscitation to decrease lactate levels [3].

However, elevated lactate levels can also originate from other causes than tissue hypoxia (e.g., inflammation, decreased lactate clearance, etc.). In patients with shock, hyperlactatemia is predominantly of hypoxic origin in the first few hours after admission, while non-hypoxic causes predominate at later stages [45]. Decreasing lactate levels also takes time, so that isolated hyperlactatemia may persist after normalization of other indices of tissue hypoperfusion [22]. Chasing lactate normalization may hence be inappropriate [23]. In patients with normalized CRT, lactate-guided resuscitation was associated with excess mortality [46]. Accordingly, monitoring of blood lactate levels has a role in assessing the effectiveness of resuscitation procedures, in conjunction with other indices of tissue hypoperfusion.

#### Echocardiography

Performing echocardiography should be considered as both left and right ventricular function may have been affected by the initial resuscitation procedures: left ventricular dysfunction may occur due to afterload increase following correction of severe hypotension; dynamic obstruction may have been caused by inotropic or vasopressor agents; and, finally, right ventricular dysfunction may be due to mechanical ventilation. In addition, sepsis-induced cardiopathy may impair the left and right ventricular function. Echocardiography may be used to evaluate volume status [34].

**Fig. 2:**
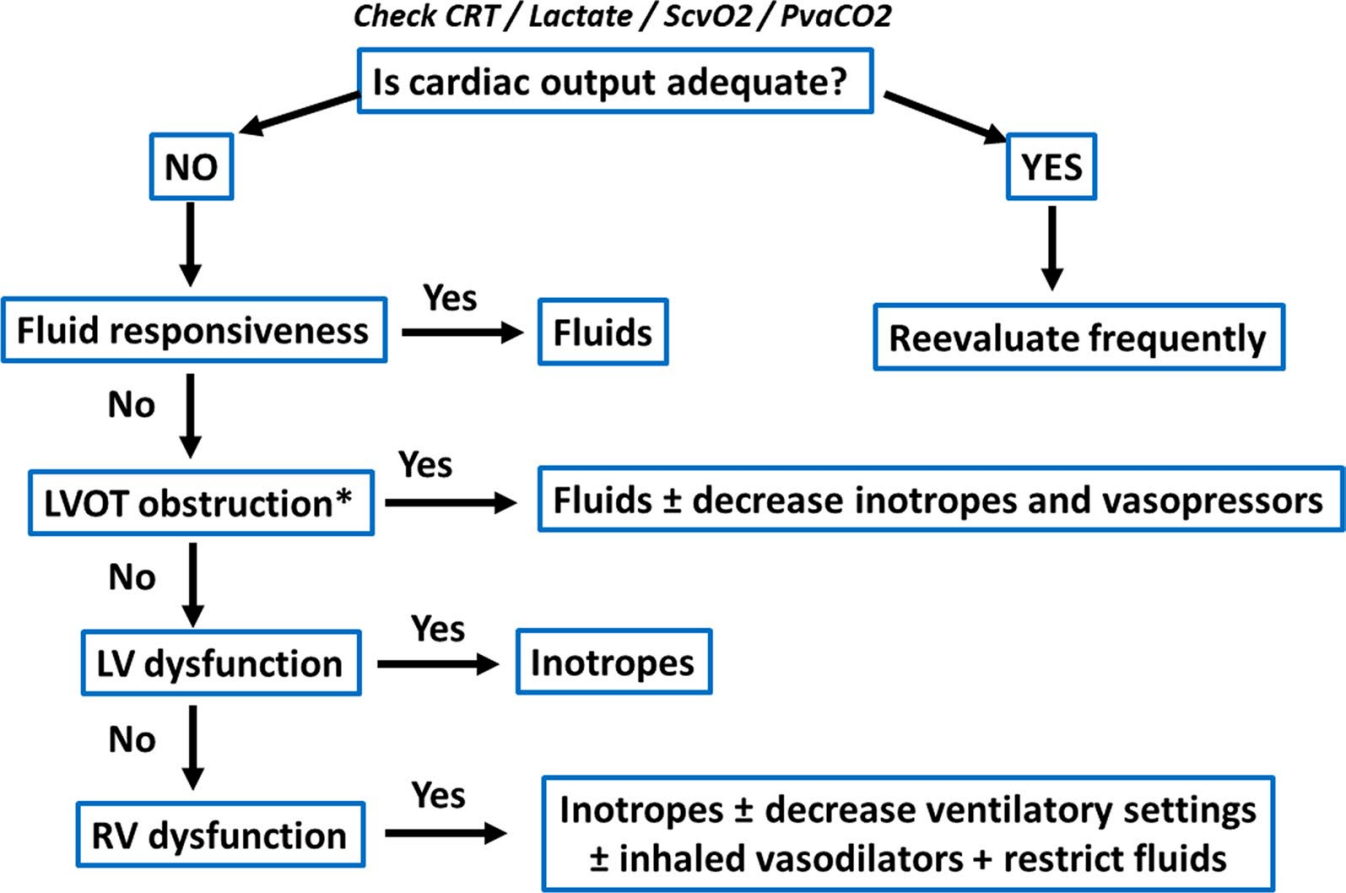
Therapeutic approach based on assessment of cardiovascular function. LVOT left ventricular outflow tract, LV left ventricle, RV right ventricle, NO nitric oxide. LVOT obstruction can only be observed with echocardiography. The other measurements can be obtained by  echocardiography as well as other monitoring techniques

In sepsis, different phenotypes can be identified by a combination of echocardiographic indices [47]. This allows fine-tuning of therapeutic interventions (Fig. 2). It is important to measure stroke volume (SV) as inotropic agents are only indicated if the impaired cardiac function is associated with a low or inadequate SV and impaired tissue perfusion. In addition, particular attention should be focused on the right ventricle, as right ventricular dysfunction may justify specific management.

#### Advanced monitoring

**Fig. 3:**
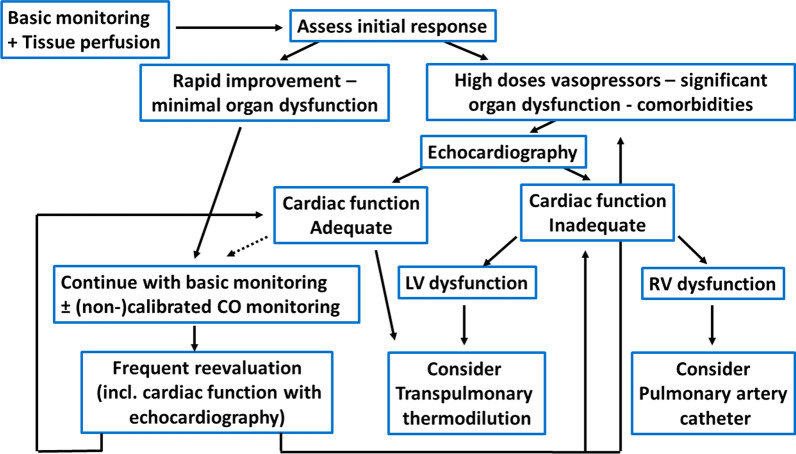
Stepwise approach for hemodynamic monitoring implementation. CO cardiac output, LV left ventricle, RV right ventricle. The dotted arrow represents an option that may be considered only in some specific cases (i.e., renal dysfunction without respiratory dysfunction)

Different tools can be used to measure CO reliably, but the choice of technique should be guided by other variables of interest, depending on patient conditions (Fig. 3). In a patient without comorbidities and with minimal organ dysfunction, non-calibrated or internally calibrated CO-monitoring devices [48] may be used but more complex patients (based on comorbidities, associated organ dysfunction or poor evolution) would benefit from the use of transpulmonary thermodilution [49] or eventually pulmonary artery catheter (PAC), coupled with echocardiography as needed [50].

Transpulmonary thermodilution (TPTD) combines a calibrated measurement of CO, a beat-by-beat and precise estimation of SV that is useful for predicting fluid responsiveness, static volumetric preload indicators, indices of cardiac function, extravascular lung water **(EVLW)** and vascular permeability. This comprehensive hemodynamic evaluation is particularly helpful in fluid management, as it provides a dynamic evaluation of fluid responsiveness coupled with an assessment of the risks associated with volume administration [34].

Through the combined measurements of CO, pulmonary pressures and SvO_2_, the use of PAC provides an assessment of the adequacy of CO and its determinants. While it does not predict fluid responsiveness, PAC is excellent for assessing fluid effectiveness and tolerance through the fluid challenge. The simultaneous measurement of CVP and pulmonary artery pressure makes PAC ideal for monitoring patients with right heart dysfunction.

Eventually, the choice of monitoring technique should be based on the patient condition, local experience and availability, and anticipated response to therapy. A stepwise approach for the patient in septic shock is suggested (Fig. 3).

### Stabilization and de-escalation: tools and indices of tolerance

During stabilization and de-escalation, the tools and targets should be adapted. During these phases, the purpose of hemodynamic support changes from optimization of tissue perfusion to prevention of complications while providing organ support. It includes evacuation of accumulated fluids and weaning of vasoactive support while preserving tissue perfusion.

During the stabilization phase, cardiac dysfunction and resuscitation-induced volume overload are common, and the already implemented hemodynamic tools often continue to be used. Lung ultrasound and the assessment of hepatic, renal, and portal venous flow patterns by ultrasound can identify signs of venous congestion which may indicate the need for volume removal and/or administration of inotropes [34]. A combination of lung/venous imaging with echocardiography is particularly useful to discriminate between volume overload and cardiac dysfunction.

A frequent complication is the development of right ventricular dysfunction that may develop in 20% of patients with acute respiratory distress syndrome (ARDS) subjected to lung protective ventilatory strategies [51]. Accordingly, repeating echocardiography at regular intervals may be helpful, especially if CVP is increasing. Managing these patients is challenging and may include the modulation of ventilatory settings and administration of inotropic agents. The choice of therapy for a given patient is based on a complex analysis of the consequences of right ventricular dysfunction on tissue perfusion, venous stasis, and ongoing need for specific ventilatory settings.

During the de-escalation phase, the situation is even more complex. Patients are usually minimally monitored, and indices of tolerance are not well defined. Should we still target the same values as in the optimization phase? Probably not, but it remains unknown how to determine the level of abnormality that may be tolerated. It sounds logical not to go back to shock (hence the term “de-resuscitation” is inappropriate). However, some degree of decrease in blood pressure and blood flow can be tolerated provided tissue oxygenation and function are preserved. Obviously, de-escalation should be stopped before hypoperfusion occurs. As an example, excessive fluid removal with ultrafiltration has been shown to be associated with increased mortality [52]. Monitoring may help to limit or prevent adverse events during de-escalation. Testing preload responsiveness before fluid removal may identify patients to whom it will be detrimental [53]. Also, measuring skin perfusion during fluid removal may identify patients in whom it will be poorly tolerated, before the onset of hypoperfusion and new onset hyperlactatemia [54]. For de-escalation of vasopressors, evaluation of dynamic arterial elastance, the ratio of pulse pressure variation to SV variation, may help to predict hypotension, and identify candidates for vasopressor reduction [55].

## Personalization of resuscitation therapies

Personalization of fluids, vasopressors, and inotropes should be tailored to tissue perfusion indices and cardiovascular state, taking into account the response to therapy.

### Personalized fluid management

Fluid management is essential in septic shock. During the salvage phase, fluids can be liberally administered as the benefits largely outweigh the risks. During optimization, the situation is more complex. The proportion of patients responding to fluids progressively decreases [13] while the likelihood of adverse events increases. Per-formula strategies, being dry or wet, are clearly inappropriate [56] and personalized strategies are preferred.

The personalized way of fluid administration comprises several steps. First, there should be a clear indication for fluids, i.e., a perfusion impairment that is expected to respond to fluids. Second, the patient should be predicted to respond to fluids. Prediction of fluid responsiveness is better achieved with dynamic tests [57] over static measurements of preload. Third, the response to fluids should be carefully evaluated using the fluid challenge technique [34].

During stabilization, resuscitation fluids, maintenance and dilution fluids should be minimized [58]. In the de-escalation phase, safe fluid withdrawal using diuretics or ultrafiltration should be achieved with minimal monitoring. First, the identification of the patient potentially benefitting from fluid removal is critical. Patients with signs of pulmonary or systemic venous stasis are ideal candidates [34]. Measurement of EVLW (by TPTD or ultrasound) can be used to trigger fluid removal and assess its efficacy. As for many other variables, the EVLW target should be individualized. As stated above, it sounds logical not to withdraw fluids in fluid-responsive patients as it may compromise their hemodynamic state [53]. Second, indices of intolerance to fluid removal should be checked. Indeed, the individual capillary refill rate is difficult to assess in clinical practice and high fluid removal rates may not be tolerated.

While guidelines suggest balanced crystalloids [3], individual factors such as chloride and albumin levels, as well as the presence of edema, should be considered when selecting between albumin and crystalloids, and between 0.9% saline and balanced crystalloids (based on chloride levels). During stabilization and de-escalation, one should also try to minimize non-resuscitation fluids and sodium load [58].

### Personalized use of vasopressors

Vasopressors are initiated, titrated, and weaned according to MAP, measures of perfusion and organ function, as mentioned above. They differ in their pharmacology, effects on capillary perfusion, organ function, and safety profiles (Table 3).

**Table 3:** Vasopressors for management of septic shock

Drug	Receptor activity	Potential biomarkers to guide treatment	Hemodynamic and organ-specific effects	Specific patient groups who may benefit
Norepinephrine	α1, α2, β1, and β3 receptor agonist		* Marked increase in MAP *Minimal increase in CO and HR	* First-line vasopressor for most patients
Dopamine	DA1 and DA2 agonist		* Moderate increase in MAP and CO * Tachycardia and arrhythmias * Impaired hypothalamic and hypophysis function	* If norepinephrine not available
Epinephrine	β-1, β-2, and α-1 receptor activity (dose-dependent)		* Marked increased in MAP and HR * Moderate increase in CO * Tachycardia and arrhythmias * Decrease in splanchnic perfusion at high doses * Major metabolic effects (acceleration of glycolysis, thermogenic effect, hypokalemia…)	* If anaphylactic component is suspected * Bradycardia septic shock * Refractory septic shock with myocardial dysfunction but the combination norepinephrine + dobutamine may be preferable (modulating doses of dobutamine according to CO)
Phenylephrine	Pure α1 agonist		* Marked increase in MAP * Decrease in CO * Decrease in splanchnic perfusion	* If norepinephrine not available
Metaraminol	α1 agonist		* Similar to low dose norepinephrine	* Short-term strategy in vasodilatory shock until more effective vasopressors available
Vasopressin	VPR1 and VPR2 agonist	LNPEP SNP angiopoetin vasopressin/copeptin	* Marked increase in MAP * Minimal increase in HR and lower incidence of arrhythmias * Minimal increase or even decrease in CO * Improvement in GFR * Impairment in splanchnic perfusion, especially at high doses * Increased risk of digital necrosis * Potential pro-aggregant effect * May improve endothelial permeability	* Patients with arrhythmias * Patients with significant AKI * Avoid in patients with peripheral ischemia *In future, identification of ideal patients using biomarkers
Terlipressin	VPR1 > > VPR2 agonist	LNPEP SNP vasopressin/copeptin	* Marked increase in MAP * Minimal increase in HR and lower incidence of arrhythmias *Minimal increase or even decrease in CO * Improvement in GFR *Impairment in splanchnic perfusion, especially at high doses *Increased risk of digital necrosis *Potential pro-aggregant effect * May improve endothelial permeability	*Patients with arrhythmias *Patients with significant AKI *Patients with cirrhosis-associated hepatorenal syndrome and systemic inflammatory syndrome * Avoid in patients with peripheral ischemia *In future, identification of ideal patients using biomarkers
Selepressin	VPR1 agonist	LNPEP SNP angiopoetin vasopressin/copeptin	* Marked increased in MAP * Minimal impact on HR and CO * Improve endothelial permeability * Impairment in splanchnic perfusion * Increased risk of myocardial ischemia	*Not available
Angiotensin II	Angiotensin I and angiotensin II receptor	ang I/ang II ratio renin AGTRAP	* Marked increase in MAP * Minimal increase in CO * Tachycardia * Improved GFR—Faster liberation from RRT * Increased risk of pulmonary embolism * Increased risk of fungal infections	* Patients with refractory septic shock * Patients receiving RRT *In future, identification of ideal patients using biomarkers

*AF* = atrial fibrillation; *AGTRAP* = angiotensin II receptor associated protein; *AKI* = acute kidney injury; *VPR* = vasopressin receptor; *DA* = dopamine; *LNPEP* leucyl and cystinyl aminopeptidase; *RCT* = randomized controlled trial; *RAAS* = renin–angiotensin–aldosterone system; *RRT* = renal replacement therapy; *SNP* = single nucleotide polymorphism; *CO* = cardiac output; *HR* = heart rate; *GFR* = glomerular filtration rate; *MAP* = mean arterial pressure

Norepinephrine is recommended as the first-line vasopressor in septic shock based on a large RCT comparing norepinephrine versus dopamine and several meta-analyses [59, 60]. In addition, the shortage of norepinephrine, and its substitution by other vasopressors, was associated with increased mortality [61]. Altogether these data confirm that norepinephrine is the first-line vasopressor. Vasopressin has been studied as a primary agent and in combination with norepinephrine [62–64]. While there was no difference in mortality, vasopressin was associated with fewer arrhythmias, lower requirement of renal replacement therapy (RRT) but a higher incidence of splanchnic and digital ischemia [65]. A similar effect was reported with terlipressin [66, 67], but no head-to-head comparison with vasopressin was performed. Selepressin administration in patients with septic shock without assessment of blood flow failed to demonstrate a beneficial impact on outcome [68], despite a favorable profile in preclinical studies [69].

Angiotensin II has emerged as a novel pressor in the treatment of vasodilatory shock. It is effective at raising blood pressure and has a catecholamine-sparing effect compared to placebo [70]. A post hoc analysis of patients receiving RRT showed improved survival and earlier liberation from RRT in patients who were randomized to angiotensin II [71].

Essential questions remain regarding optimal vasopressor selection, the role of combination therapy, and the most effective and safest method of escalation in different patient cohorts [72]. More tools are needed to inform clinicians about the most effective vasopressor in particular settings and how to avoid harm.

Several genetic polymorphisms are associated with different responses to vasopressor agents [73, 74] but this targeted strategy is not yet suitable for bedside use. Alternatively, biomarkers may be used to indicate which cohort of patients may benefit more from a particular agent. Plasma angiopoietin 1 (Ang1) and angiopoietin 2 (Ang2), mediators of vascular permeability, have emerged as potential biomarkers to guide vasopressin therapy. Serum renin and the angiotensin I/II ratio reflect the activity of angiotensin-converting enzymes [75] and are promising biomarkers to identify patients with vasodilatory shock for whom treatment with angiotensin II may be beneficial [76]. Confirmation in prospective studies is needed.

Finally, the evidence regarding the optimal therapeutic strategy for shock requiring high-dose vasopressors is scant. The α1 receptors, like any other vasopressor receptor, may be saturated and/or hypo-responsive in refractory shock. It makes sense to consider alternative vasopressors that act on different receptors (vasopressin derivatives or angiotensin II) rather than using an agent from the same class.

Similarly, the indications for adjunctive therapies vary and predictive tools are necessary. Identification of patients responding to corticosteroids may be helped by the analysis of selected cytokines [77] or identification of specific transcriptomic endotypes [78]. In the absence of timely implementation of these techniques, hydrocortisone is nowadays considered in patients receiving high doses of vasopressor agents.

## Personalized use of inotropic agents

There are no data to support the systematic use of inotropic agents. The indiscriminate use of levosimendan in such patients was unsuccessful [79]. Even attempting to identify patients with myocardial injury based on biomarkers was not helpful [80]. This is not surprising since the consequences of sepsis-associated myocardial depression are highly variable. Some patients present with a high CO [47] or even dynamic obstruction of the outflow tract [81] despite significant impairment in cardiac function or high levels of biomarkers. They should not be treated with inotropic agents. On the other hand, some patients present with a low CO related to left or right ventricular dysfunction [47] and may benefit from inotropic agents. Accordingly, as stated above, inotropes may be indicated only in patients with signs of tissue hypoperfusion related to a low CO induced by impaired cardiac function. Adverse effects (tachycardia, arrhythmias) and specific risks in some patient categories (hypertrophic cardiomyopathies, myocardial ischemia) should be cautiously scrutinized, and risks/benefits of the intervention evaluated.

Discussion on the type of agent is more complex. No agent has proven to be superior to another in patients in shock. Of note, most trials comparing inotropic agents were performed in patients with heart failure and excluded patients in cardiogenic shock. Accordingly, meta-analyses of these trials should be considered cautiously.

Based on pharmacologic properties, we suggest the following stepwise approach: First, test a limited dose of dobutamine (2.5 to 5 mcg/kg/min) and evaluate efficacy and tolerance. In cases of severe contractility impairment, higher doses (up to 20 mcg/kg/min) may be considered. Second, substitute or add enoximone or milrinone and evaluate efficacy and tolerance (beware of the risk of hypotension). Third, substitute or add levosimendan in cases of severe impairment. At each step, efficacy (improvement in cardiac function and CO, resolution of tissue hypoperfusion) and tolerance (e.g., lack of tachycardia, arrhythmias, etc.) should be evaluated. For each of the agents, the lowest dose associated with the desired effect should be administered. As soon as the situation improves, weaning of inotropes should be attempted.

## Conclusions

Even though some general rules apply for septic shock management, a personalized approach should be considered for hemodynamic monitoring and support. Importantly, monitoring and support should be adapted to the four stages of shock and the impact of the interventions should be continuously evaluated.

## Data Availability

N/A, no new data were generated.

## References

[1] Cecconi M, De Backer D, Antonelli M, Beale RJ, Bakker J, Hofer C, Jaeschke R, Mebazaa A, Pinsky MR, Teboul JL (2014). Consensus on circulatory shock and hemodynamic monitoring. Task force of the European Society of Intensive Care Medicine. Intensive Care Med.

[2] Vincent JL, De Backer D (2013). Circulatory shock. N Engl J Med.

[3] Evans L, Rhodes A, Alhazzani W, Antonelli M, Coopersmith CM, French C, Machado FR, McIntyre L, Ostermann M, Prescott HC (2021). Surviving sepsis campaign: international guidelines for management of sepsis and septic shock 2021. Intensive Care Med.

[4] Vincent JL, Singer M, Einav S, Moreno R, Wendon J, Teboul JL, Bakker J, Hernandez G, Annane D, de Man AME (2021). Equilibrating SSC guidelines with individualized care. Crit Care.

[5] Coopersmith CM, De Backer D, Deutschman CS, Ferrer R, Lat I, Machado FR, Martin GS, Martin-Loeches I, Nunnally ME, Antonelli M (2018). Surviving sepsis campaign: research priorities for sepsis and septic shock. Intensive Care Med.

[6] Ait-Oufella H, Bige N, Boelle PY, Pichereau C, Alves M, Bertinchamp R, Baudel JL, Galbois A, Maury E, Guidet B (2014). Capillary refill time exploration during septic shock. Intensive Care Med.

[7] Ait-Oufella H, Lemoinne S, Boelle PY, Galbois A, Baudel JL, Lemant J, Joffre J, Margetis D, Guidet B, Maury E (2011). Mottling score predicts survival in septic shock. Intensive Care Med.

[8] De Backer D, Vieillard-Baron A (2019). Clinical examination: a trigger but not a substitute for hemodynamic evaluation. Intensive Care Med.

[9] Zieleskiewicz L, Lopez A, Hraiech S, Baumstarck K, Pastene B, Di Bisceglie M, Coiffard B, Duclos G, Boussuges A, Bobbia X (2021). Bedside POCUS during ward emergencies is associated with improved diagnosis and outcome: an observational, prospective, controlled study. Crit Care.

[10] Teboul JL, Saugel B, Cecconi M, De Backer D, Hofer CK, Monnet X, Perel A, Pinsky MR, Reuter DA, Rhodes A (2016). Less invasive hemodynamic monitoring in critically ill patients. Intensive Care Med.

[11] Bréchot N, Hajage D, Kimmoun A, Demiselle J, Agerstrand C, Montero S, Schmidt M, Luyt CE, Lebreton G, Hékimian G (2020). Venoarterial extracorporeal membrane oxygenation to rescue sepsis-induced cardiogenic shock: a retrospective, multicentre, international cohort study. Lancet.

[12] Cecconi M, Hernandez G, Dunser M, Antonelli M, Baker T, Bakker J, Duranteau J, Einav S, Groeneveld ABJ, Harris T (2019). Fluid administration for acute circulatory dysfunction using basic monitoring: narrative review and expert panel recommendations from an ESICM task force. Intensive Care Med.

[13] Hernandez G, Ospina-Tascon GA, Damiani LP, Estenssoro E, Dubin A, Hurtado J, Friedman G, Castro R, Alegria L, Teboul JL (2019). Effect of a resuscitation strategy targeting peripheral perfusion status vs serum lactate levels on 28-day mortality among patients with septic shock: the ANDROMEDA-SHOCK randomized clinical trial. JAMA.

[14] Ait-Hamou Z, Teboul JL, Anguel N, Monnet X (2019). How to detect a positive response to a fluid bolus when cardiac output is not measured?. Ann Intensive Care.

[15] Jacquet-Lagreze M, Bouhamri N, Portran P, Schweizer R, Baudin F, Lilot M, Fornier W, Fellahi JL (2019). Capillary refill time variation induced by passive leg raising predicts capillary refill time response to volume expansion. Crit Care.

[16] Vincent JL, Nielsen ND, Shapiro NI, Gerbasi ME, Grossman A, Doroff R, Zeng F, Young PJ, Russell JA (2018). Mean arterial pressure and mortality in patients with distributive shock: a retrospective analysis of the MIMIC-III database. Ann Intensive Care.

[17] Persichini R, Silva S, Teboul JL, Jozwiak M, Chemla D, Richard C, Monnet X (2012). Effects of norepinephrine on mean systemic pressure and venous return in human septic shock. Crit Care Med.

[18] Ospina-Tascon GA, Hernandez G, Alvarez I, Calderon-Tapia LE, Manzano-Nunez R, Sanchez-Ortiz AI, Quinones E, Ruiz-Yucuma JE, Aldana JL, Teboul JL (2020). Effects of very early start of norepinephrine in patients with septic shock: a propensity score-based analysis. Crit Care.

[19] Ospina-Tascón GA, Teboul JL, Hernandez G, Alvarez I, Sánchez-Ortiz AI, Calderón-Tapia LE, Manzano-Nunez R, Quiñones E, Madriñan-Navia HJ, Ruiz JE (2020). Diastolic shock index and clinical outcomes in patients with septic shock. Ann Intensive Care.

[20] De Backer D, Creteur J, Noordally O, Smail N, Gulbis B, Vincent JL (1998). Does hepato-splanchnic VO2/DO2 dependency exist in critically ill septic patients?. Am J Respir Crit Care Med.

[21] De Backer D, Creteur J, Preiser JC, Dubois MJ, Vincent JL (2002). Microvascular blood flow is altered in patients with sepsis. Am J Respir Crit Care Med.

[22] Hernandez G, Luengo C, Bruhn A, Kattan E, Friedman G, Ospina-Tascon GA, Fuentealba A, Castro R, Regueira T, Romero C (2014). When to stop septic shock resuscitation: clues from a dynamic perfusion monitoring. Ann Intensive Care.

[23] Bakker J, De Backer D, Hernandez G (2016). Lactate-guided resuscitation saves lives: we are not sure. Intensive Care Med.

[24] Brunauer A, Koköfer A, Bataar O, Gradwohl-Matis I, Dankl D, Bakker J, Dünser MW (2016). Changes in peripheral perfusion relate to visceral organ perfusion in early septic shock: a pilot study. J Crit Care.

[25] Boerma EC, Kuiper MA, Kingma WP, Egbers PH, Gerritsen RT, Ince C (2008). Disparity between skin perfusion and sublingual microcirculatory alterations in severe sepsis and septic shock: a prospective observational study. Intensive Care Med.

[26] Brabrand M, Hosbond S, Folkestad L (2011). Capillary refill time: a study of interobserver reliability among nurses and nurse assistants. Eur J Emerg Med.

[27] Zampieri FG, Damiani LP, Bakker J, Ospina-Tascon GA, Castro R, Cavalcanti AB, Hernandez G (2019). Effect of a resuscitation strategy targeting peripheral perfusion status vs serum lactate levels on 28-day mortality among patients with septic shock: a Bayesian Reanalysis of the ANDROMEDA-SHOCK Trial. Am J Respir Crit Care Med.

[28] Liu X, Luo D, Zhang J, Du L (2020). Distribution and relative expression of vasoactive receptors on arteries. Sci Rep.

[29] Asfar P, Meziani F, Hamel JF, Grelon F, Megarbane B, Anguel N, Mira JP, Dequin PF, Gergaud S, Weiss N (2014). High versus low blood-pressure target in patients with septic shock. N Engl J Med.

[30] Lamontagne F, Richards-Belle A, Thomas K, Harrison DA, Sadique MZ, Grieve RD, Camsooksai J, Darnell R, Gordon AC, Henry D (2020). Effect of reduced exposure to vasopressors on 90-day mortality in older critically Ill patients with vasodilatory hypotension: a randomized clinical trial. JAMA.

[31] Deruddre S, Cheisson G, Mazoit JX, Vicaut E, Benhamou D, Duranteau J (2007). Renal arterial resistance in septic shock: effects of increasing mean arterial pressure with norepinephrine on the renal resistive index assessed with Doppler ultrasonography. Intensive Care Med.

[32] Gershengorn HB, Stelfox HT, Niven DJ, Wunsch H (2020). Association of premorbid blood pressure with vasopressor infusion duration in patients with shock. Am J Respir Crit Care Med.

[33] De Backer D, Vincent JL (2018). Should we measure the central venous pressure to guide fluid management? Ten answers to 10 questions. Crit Care.

[34] De Backer D, Aissaoui N, Cecconi M, Chew MS, Denault A, Hajjar L, Hernandez G, Messina A, Myatra SN, Ostermann M (2022). How can assessing hemodynamics help to assess volume status?. Intensive Care Med.

[35] De Backer D, Creteur J, Dubois MJ, Sakr Y, Vincent JL (2004). Microvascular alterations in patients with acute severe heart failure and cardiogenic shock. Am Heart J.

[36] De Backer D, Vincent JL (2016). Early goal-directed therapy: do we have a definitive answer?. Intensive Care Med.

[37] Ospina-Tascon GA, Umana M, Bermudez WF, Bautista-Rincon DF, Valencia JD, Madrinan HJ, Hernandez G, Bruhn A, Arango-Davila C, De Backer D (2016). Can venous-to-arterial carbon dioxide differences reflect microcirculatory alterations in patients with septic shock?. Intensive Care Med.

[38] Vallee F, Vallet B, Mathe O, Parraguette J, Mari A, Silva S, Samii K, Fourcade O, Genestal M (2008). Central venous-to-arterial carbon dioxide difference: an additional target for goal-directed therapy in septic shock?. Intensive Care Med.

[39] Mesquida J, Saludes P, Gruartmoner G, Espinal C, Torrents E, Baigorri F, Artigas A (2015). Central venous-to-arterial carbon dioxide difference combined with arterial-to-venous oxygen content difference is associated with lactate evolution in the hemodynamic resuscitation process in early septic shock. Crit Care.

[40] Ospina-Tascon GA, Bautista-Rincon DF, Umana M, Tafur JD, Gutierrez A, Garcia AF, Bermudez W, Granados M, Arango-Davila C, Hernandez G (2013). Persistently high venous-to-arterial carbon dioxide differences during early resuscitation are associated with poor outcomes in septic shock. Crit Care.

[41] Du W, Liu DW, Wang XT, Long Y, Chai WZ, Zhou X, Rui X (2013). Combining central venous-to-arterial partial pressure of carbon dioxide difference and central venous oxygen saturation to guide resuscitation in septic shock. J Crit Care.

[42] Ospina-Tascon GA, Umana M, Bermudez W, Bautista-Rincon DF, Hernandez G, Bruhn A, Granados M, Salazar B, Arango-Davila C, De Backer D (2015). Combination of arterial lactate levels and venous-arterial CO to arterial-venous O content difference ratio as markers of resuscitation in patients with septic shock. Intensive Care Med.

[43] Monnet X, Julien F, Ait-Hamou N, Lequoy M, Gosset C, Jozwiak M, Persichini R, Anguel N, Richard C, Teboul JL (2013). Lactate and venoarterial carbon dioxide difference/arterial-venous oxygen difference ratio, but not central venous oxygen saturation, predict increase in oxygen consumption in fluid responders. Crit Care Med.

[44] Jansen TC, van Bommel J, Schoonderbeek J, Sleeswijk Visser SJ, van der Klooster JM, Lima AP, Willemsen SP, Bakker J (2010). Early lactate-guided therapy in ICU patients: a multicenter, open-label, randomized, controlled trial. Am J Respir Crit Care Med.

[45] Rimachi R, Bruzzi DC, Orellano-Jimenez C, Cotton F, Vincent J, De Backer D (2012). Lactate/pyruvate ratio as a marker of tissue hypoxia in circulatory and septic shock. Anaesth Intensive Care.

[46] Kattan E, Hernández G, Ospina-Tascón G, Valenzuela ED, Bakker J, Castro R (2020). A lactate-targeted resuscitation strategy may be associated with higher mortality in patients with septic shock and normal capillary refill time: a post hoc analysis of the ANDROMEDA-SHOCK study. Ann Intensive Care.

[47] Geri G, Vignon P, Aubry A, Fedou AL, Charron C, Silva S, Repessé X, Vieillard-Baron A (2019). Cardiovascular clusters in septic shock combining clinical and echocardiographic parameters: a post hoc analysis. Intensive Care Med.

[48] Kouz K, Scheeren TWL, de Backer D, Saugel B (2021). Pulse wave analysis to estimate cardiac output. Anesthesiology.

[49] Monnet X, Teboul JL (2017). Transpulmonary thermodilution: advantages and limits. Crit Care.

[50] Teboul JL, Saugel B, Cecconi M, De BD, Hofer CK, Monnet X, Perel A, Pinsky MR, Reuter DA, Rhodes A (2016). Less invasive hemodynamic monitoring in critically ill patients. Intensive Care Med.

[51] Boissier F, Katsahian S, Razazi K, Thille AW, Roche-Campo F, Leon R, Vivier E, Brochard L, Vieillard-Baron A, Brun-Buisson C (2013). Prevalence and prognosis of cor pulmonale during protective ventilation for acute respiratory distress syndrome. Intensive Care Med.

[52] Murugan R, Kerti SJ, Chang CH, Gallagher M, Clermont G, Palevsky PM, Kellum JA, Bellomo R (2019). Association of net ultrafiltration rate with mortality among critically Ill adults with acute kidney injury receiving continuous venovenous hemodiafiltration: a secondary analysis of the randomized evaluation of normal vs augmented level (RENAL) of renal replacement therapy trial. JAMA Netw Open.

[53] Monnet X, Cipriani F, Camous L, Sentenac P, Dres M, Krastinova E, Anguel N, Richard C, Teboul JL (2016). The passive leg raising test to guide fluid removal in critically ill patients. Ann Intensive Care.

[54] Mongkolpun W, Bakos P, Vincent JL, Creteur J (2021). Monitoring skin blood flow to rapidly identify alterations in tissue perfusion during fluid removal using continuous veno-venous hemofiltration in patients with circulatory shock. Ann Intensive Care.

[55] Guinot PG, Bernard E, Levrard M, Dupont H, Lorne E (2015). Dynamic arterial elastance predicts mean arterial pressure decrease associated with decreasing norepinephrine dosage in septic shock. Crit Care.

[56] Meyhoff TS, Hjortrup PB, Wetterslev J, Sivapalan P, Laake JH, Cronhjort M, Jakob SM, Cecconi M, Nalos M, Ostermann M (2022). Restriction of intravenous fluid in ICU patients with septic shock. N Engl J Med.

[57] Monnet X, Shi R, Teboul JL (2022). Prediction of fluid responsiveness. What's new?. Annal Intensive Care.

[58] Van Regenmortel N, Verbrugghe W, Roelant E, Van den Wyngaert T, Jorens PG (2018). Maintenance fluid therapy and fluid creep impose more significant fluid, sodium, and chloride burdens than resuscitation fluids in critically ill patients: a retrospective study in a tertiary mixed ICU population. Intensive Care Med.

[59] De Backer D, Biston P, Devriendt J, Madl C, Chochrad D, Aldecoa C, Brasseur A, Defrance P, Gottignies P, Vincent JL (2010). Comparison of dopamine and norepinephrine in the treatment of shock. N Engl J Med.

[60] De Backer D, Aldecoa C, Njimi H, Vincent J-L (2012). Dopamine versus norepinephrine in the treatment of septic shock: a metaanalysis. Crit Care Med.

[61] Vail E, Gershengorn HB, Hua M, Walkey AJ, Rubenfeld G, Wunsch H (2017). Association between US norepinephrine shortage and mortality among patients with septic shock. JAMA.

[62] Russell JA, Walley KR, Singer J, Gordon AC, Hebert PC, Cooper DJ, Holmes CL, Mehta S, Granton JT, Storms MM (2008). Vasopressin versus norepinephrine infusion in patients with septic shock. N Engl J Med.

[63] Gordon AC, Mason AJ, Thirunavukkarasu N, Perkins GD, Cecconi M, Cepkova M, Pogson DG, Aya HD, Anjum A, Frazier GJ (2016). Effect of early vasopressin vs norepinephrine on kidney failure in patients with septic shock: The VANISH randomized clinical trial. JAMA.

[64] Hajjar LA, Vincent JL, Barbosa Gomes Galas FR, Rhodes A, Landoni G, Osawa EA, Melo RR, Sundin MR, Grande SM, Gaiotto FA (2017). Vasopressin versus norepinephrine in patients with vasoplegic shock after cardiac surgery: the VANCS randomized controlled trial. Anesthesiology.

[65] Nagendran M, Russell JA, Walley KR, Brett SJ, Perkins GD, Hajjar L, Mason AJ, Ashby D, Gordon AC (2019). Vasopressin in septic shock: an individual patient data meta-analysis of randomised controlled trials. Intensive Care Med.

[66] Liu ZM, Chen J, Kou Q, Lin Q, Huang X, Tang Z, Kang Y, Li K, Zhou L, Song Q (2018). Terlipressin versus norepinephrine as infusion in patients with septic shock: a multicentre, randomised, double-blinded trial. Intensive Care Med.

[67] Zhu Y, Huang H, Xi X, Du B (2019). Terlipressin for septic shock patients: a meta-analysis of randomized controlled study. J Intensive Care.

[68] Laterre PF, Berry SM, Blemings A, Carlsen JE, Francois B, Graves T, Jacobsen K, Lewis RJ, Opal SM, Perner A (2019). Effect of selepressin vs placebo on ventilator- and vasopressor-free days in patients with septic shock: the SEPSIS-ACT randomized clinical trial. JAMA.

[69] Rehberg S, Ertmer C, Vincent JL, Morelli A, Schneider M, Lange M, Van Aken H, Traber DL, Westphal M (2011). Role of selective V1a receptor agonism in ovine septic shock. Crit Care Med.

[70] Khanna A, English SW, Wang XS, Ham K, Tumlin J, Szerlip H, Busse LW, Altaweel L, Albertson TE, Mackey C (2017). Angiotensin II for the treatment of vasodilatory shock. N Engl J Med.

[71] Tumlin JA, Murugan R, Deane AM, Ostermann M, Busse LW, Ham KR, Kashani K, Szerlip HM, Prowle JR, Bihorac A (2018). Outcomes in patients with vasodilatory shock and renal replacement therapy treated with intravenous angiotensin II. Crit Care Med.

[72] Chawla LS, Ostermann M, Forni L, Tidmarsh GF (2019). Broad spectrum vasopressors: a new approach to the initial management of septic shock?. Crit Care.

[73] Nakada TA, Russell JA, Boyd JH, Aguirre-Hernandez R, Thain KR, Thair SA, Nakada E, McConechy M, Walley KR (2010). beta2-Adrenergic receptor gene polymorphism is associated with mortality in septic shock. Am J Respir Crit Care Med.

[74] Anantasit N, Boyd JH, Walley KR, Russell JA (2014). Serious adverse events associated with vasopressin and norepinephrine infusion in septic shock. Crit Care Med.

[75] Chawla LS, Chen S, Bellomo R, Tidmarsh GF (2018). Angiotensin converting enzyme defects in shock: implications for future therapy. Crit Care.

[76] Bellomo R, Forni LG, Busse LW, McCurdy MT, Ham KR, Boldt DW, Hästbacka J, Khanna AK, Albertson TE, Tumlin J (2020). Renin and survival in patients given angiotensin II for catecholamine-resistant vasodilatory shock. A clinical trial. Am J Respir Crit Care Med.

[77] Bentzer P, Fjell C, Walley KR, Boyd J, Russell JA (2016). Plasma cytokine levels predict response to corticosteroids in septic shock. Intensive Care Med.

[78] Antcliffe DB, Burnham KL, Al-Beidh F, Santhakumaran S, Brett SJ, Hinds CJ, Ashby D, Knight JC, Gordon AC (2019). Transcriptomic signatures in sepsis and a differential response to steroids. From the VANISH randomized trial. Am J Respir Crit Care Med.

[79] Gordon AC, Perkins GD, Singer M, McAuley DF, Orme RM, Santhakumaran S, Mason AJ, Cross M, Al-Beidh F, Best-Lane J (2016). Levosimendan for the prevention of acute organ dysfunction in sepsis. N Engl J Med.

[80] Antcliffe DB, Santhakumaran S, Orme RML, Ward JK, Al-Beidh F, O'Dea K, Perkins GD, Singer M, McAuley DF, Mason AJ (2019). Levosimendan in septic shock in patients with biochemical evidence of cardiac dysfunction: a subgroup analysis of the LeoPARDS randomised trial. Intensive Care Med.

[81] Chauvet JL, El-Dash S, Delastre O, Bouffandeau B, Jusserand D, Michot JB, Bauer F, Maizel J, Slama M (2015). Early dynamic left intraventricular obstruction is associated with hypovolemia and high mortality in septic shock patients. Crit Care.

